# Real-Life Analysis with Erenumab: First Target Therapy in the Episodic and Chronic Migraine’s Prophylaxis

**DOI:** 10.3390/jcm10194425

**Published:** 2021-09-27

**Authors:** Zaira Maraia, Diletta Ricci, Marco Bruno Luigi Rocchi, Alessandro Moretti, Celestino Bufarini, Arturo Cavaliere, Manola Peverini

**Affiliations:** 1 Biomolecular Sciences Department, University of Urbino, 61029 Urbino, Italy; z.maraia@campus.uniurb.it (Z.M.); d.ricci11@campus.uniurb.it (D.R.); marco.rocchi@uniurb.it (M.B.L.R.); 2 Neurological Centre for Cognitive Disorders and Dementia, ASUR Marche AV1, 61034 Fossombrone, Italy; alessandro.moretti@sanita.marche.it; 3 Urbino Hospital Pharmacy, ASUR Marche AV1, 61029 Urbino, Italy; celestino.bufarini@sanita.marche.it; 4 Local Pharmaceutical Service, Viterbo Local Health Authority, 01100 Viterbo, Italy; arturo.cavaliere@asl.vt.it

**Keywords:** Erenumab, migraine, real-life analysis, CGRP, MIDAS

## Abstract

Background: to research retrospectively the efficacy of Erenumab’s treatment, thus allowing to describe a summary more in line with the reality observed every day in clinical practice, relative to a sample of patients widely heterogeneous. The study aims to confirm the efficacy of Erenumab, in terms of reduction of migraine days per month, from baseline to month 12 of treatment. Additional objectives included a reduction in the number of days of symptomatic drug use and change from baseline in the Migraine Disability Assessment Score Questionnaire (MIDAS); Methods: the analysis included all patients treated for 12 months with Erenumab during the year 2019–2020. The population analyzed consists of twenty-six patients from the Neurology outpatient clinic in Fossombrone. Several quantitative and qualitative variables were recorded by reading the medical records of the patients. The MIDAS was administered to patients to assess the disability related to migraine; Results: at the end of treatment, a statistically significant reduction in the mean number of monthly migraine days, acute medication use per month, and MIDAS questionnaire score was observed; Conclusions: as a preventive treatment of episodic and chronic migraine, our analysis data confirm the efficacy of Erenumab for the prevention of the migraine. The success is achieved in 96% of cases.

## 1. Introduction

Migraine is the third most prevalent and the second most disabling disease worldwide. Migraine is characterized by recurrent headache episodes of moderate to severe intensity that last 4–72 h associated with neuro-vegetative symptoms [1]. Migraine tends to be sometimes considered of little importance because it is a condition that does not lead to a reduction in life expectancy. Migraine is ranked second among causes of disability according to the Global Burden of Disease Study 2019 (GBD) [2]. Approximately 11.6% of the Italian population is affected by migraines. Migraine is two to three times more prevalent in women than men, but for both sexes, the highest incidence is between 30 and 39 years old, the period of highest work and social productivity of the individual [3,4]. In the past, migraine was classically understood as a vascular disease caused by vasoconstriction and the subsequent prompt vasodilatory response of the cerebral vessels. However, advanced imaging techniques have demonstrated primary neuronal involvement in the pathophysiology of migraines. Migraine pain is underlined by a dysfunctional trigeminal system, which consists of the Central Nervous System (CNS) and peripheral structures. The calcitonin gene-related peptide (GCRP) is the key neurotransmitter involved in these areas. GCRP acts through a metabotropic Gs protein-associated receptor, thus playing important roles both peripherally and centrally. Specifically, a prominent peripheral effect is dilation of the vascular beds, particularly in intra- and extracranial arteries, but even more important is its ability to induce mast cells degranulation. Persistent activation of peripheral nociceptors combined with triggering of pro-inflammatory mechanisms leads to peripheral sensitization. On the other hand, CGRP is a neuromodulator capable of increasing glutamatergic transmission leading to central sensitization [5,6,7]. As far as a pharmacological treatment is concerned, two mechanisms of action can be suggested independently or in parallel, depending on the severity of the disease. Triptans and Non-Steroidal Anti-Inflammatory drugs (NSAIDs) are suggested for acute attack treatment, whereas a prophylactic therapy with various pharmacological classes, including antiepileptics, tricyclic antidepressants, b-blockers, calcium channel blockers, and botulinum toxin is generally applied [8]. Despite the availability of these different therapeutic options, which were not specifically developed for migraine prophylaxis, patients have numerous unmet therapeutic needs. This is because these therapies are associated with significant side effects that, together with the frequency of administration, poor results and not less than direct and indirect health care costs affect compliance and adherence. All of this constitutes a significant limitation for the management of patients with migraine pain and represents a risk for medication overuse and possible abuse of symptomatic drugs with chronicity of the disease [9,10]. Erenumab is the new first target therapy for the prevention of migraines after about 20 years of absence of a new therapy. It is a human monoclonal antibody, a highly selective antagonist of the CGRP receptor, with a half-life of about 28 days that allows a monthly subcutaneous injection. Thus, blocking the interaction of CGRP with its receptor interrupts the signal to the trigeminal system and prevents the perception of migraine pain and all its associated symptom [11,12,13].

## 2. Materials and Methods

All patients with episodic and chronic migraine, with or without aura, were included in the observational analysis, during the year 2019–2020 and treated with Erenumab for 1 year at the Neurology Outpatient Clinic of the Fossombrone Hospital. As the drug had already been authorized by the Italian Medicines Agency (AIFA) Technical and Scientific Committee (CTS) at the time but was still under negotiation by the national Price and Reimbursement Commission (CPR), provision was made for facilitated access to the drug, which AIFA grants in special cases where it is necessary to satisfy a therapeutic need that cannot be met by drugs already on the market. In this case, it was a non-negotiated class C pathway (CNN), and at the hospital level, a procedure characterized by eligibility criteria, monitored cycles of therapy and follow-up measurements were envisaged. As there were no reimbursement criteria or prescribing rules yet, the drug was supplied by a simple request from the center itself. Before starting treatment, all patients had to present at least 4 migraine days per month and show an insufficient response or intolerance to at least 2 classes of migraine prophylaxis drugs, according to the established eligibility criteria. Erenumab was administered preliminarily by the clinician, during the outpatient visit. Patients were allowed to use concomitant preventive treatments along with the use of drugs for the acute attack at the first prodromal manifestations. Approximately every three months, patients were monitored by the clinician with an in-depth interview to assess clinical parameters such as the number of migraine days per month, use of symptomatic drugs, and headache-related disability. For all patients were recorded on Excel, by reading the clinical cards, gender, age, year of diagnosis and history, days of migraine per month before treatment with Erenumab, family history, and comorbidities. Previous prophylactic therapy and the score of the MIDAS questionnaire administered by the clinician at baseline and after 3 months of treatment were also recorded. To limit as much as possible, the spread of contagion, a telephone interview was carried out for the Fossombrone Centre, through which the MIDAS questionnaire was administered. The questionnaire is a representative control tool for the 12th month of treatment with Erenumab. The MIDAS questionnaire, with proven validity and reliability, is an important tool for understanding how migraines can negatively impact the lives of patients [14]. The questionnaire consists of 5 questions assessing days of no or substantially reduced activity in three domains: school/work, home, and leisure time. The final score is obtained by simply adding up the values reported for each answer; this allows the patient to be placed in one of the 4 degrees of disability related to headache:

(1) Grade I, minimal or negligible disability, score from 0 to 5;
(2) Grade II, mild disability, a score from 6 to 11;
(3) Grade III, average disability, a score of 11 to 20;
(4) Grade IV, severe disability, a score of 21 or more.

The data collected, were subjected to statistical analysis. First of all, the data were analyzed using two types of statistical analysis: uni- and multivariate analyses. First of all, a descriptive analysis was carried out to give a synthetic representation of the results of the observations. The indices used in this analysis are central tendency indices such as the Mean and dispersion indices such as the Standard Deviation. For qualitative variables, absolute and relative frequencies were analyzed. Differences between averages for quantitative variables were assessed using paired t-tests and 95% CIs. McNemar’s non-parametric test was used to test for differences in data recoded into dichotomous variables (conversion from chronic to episodic migraine and conversion from medication overuse to non-medication overuse) and therefore for 2 × 2 contingency tables. Finally, multivariate logistic regression (logit model) was used to estimate the probability that subjects belonged to the super responder category as a function of the other variables under observation. This model is a non-linear regression model used when the dependent variable is dichotomous and expresses the log of the ODD, therefore the ratio between the probability of success (being a super responder) and the probability of failure (not being a super responder). Statistical processing of the results was carried out using Statistical Package for Social Science (SPSS) software, version 23.0 IBM SPSS Company, Armonk, NY, USA, 2018.

Purely observational studies such as real-life analysis do not require registration in Italy. Furthermore, the University Ethical Committee of Urbino didn’t assess our analysis as it wasn’t considered of ethical importance.

## 3. Results

The general characteristics of the study sample are summarized in Table 1. As can be seen, the prevalence of migraine in women compared to men is consistent with the national data, which confirms that migraine is a predominant disorder in women [15].

The analysis according to the age variable shows the average age of 51 years (SD = 11.01). All patients had at least 3 failures with preventive treatment leading to discontinuation of therapy, due to no significant improvement or adverse events (Table 2).

With regard to the various comorbidities encountered in clinical practice, psychiatric ones are generally of considerable importance [16]. The results show that 71% of the patients suffered from disorders such as anxiety and/or depression before treatment with Erenumab. This is significant because if migraine occurs in combination with depression and anxiety, patients tend to suffer more severe migraine attacks, show a poor response to conventional preventive treatments, and have an increased risk of medication overuse. Table 3 summarizes the mean values of the three clinical parameters used to observe the effects of Erenumab on a population of patients with chronic and episodic migraines.

**Figure 1:**
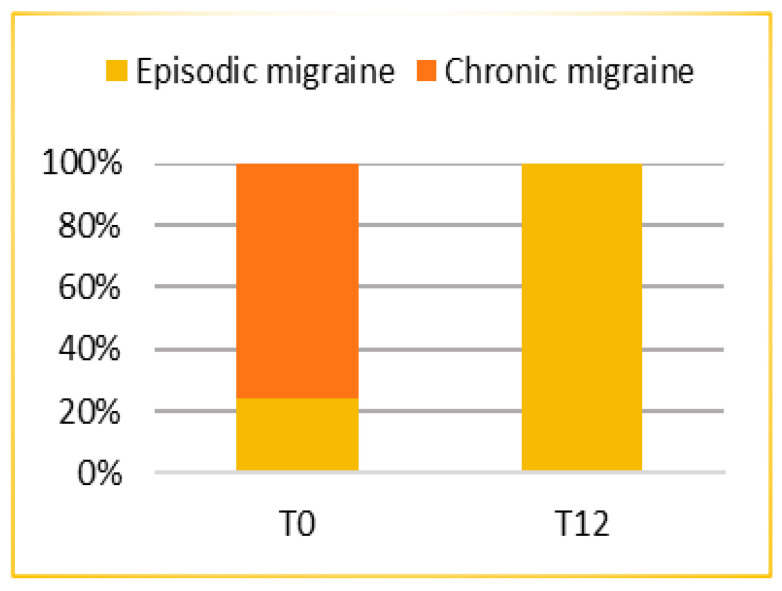
Conversion from chronic to episodic migraine after 12 months of treatment.

The analysis showed that after 1 year of treatment (T12) the mean number of migraine days per month (MMD) was 3.36 (SD = 2.413; ES = 0.482), thus patients reported a statistically significant (*p* < 0.0005) mean reduction of 11.88 days from baseline (T0) (95% CI: 10.06; 13.70). A highly and statistically significant (*p* < 0.0005) conversion of migraine from chronic to episodic is reported in Figure 1.

**Figure 2:**
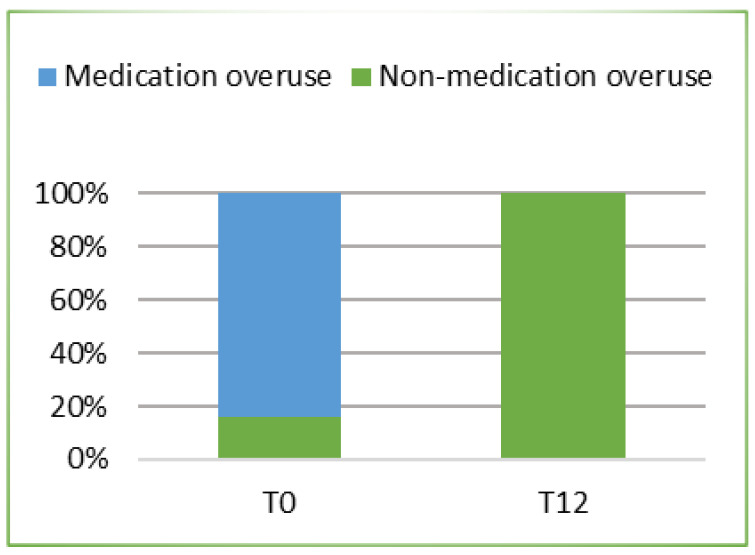
The proportion of patients with medication overuse at baseline who changed status to non–medication overuse.

In parallel with the reduction in the number of days with migraine, treatment with Erenumab was also effective in reducing the number of days of consumption of symptomatic drugs. In fact, after 12 months of treatment, the mean number of days of use of these drugs was 2.6 (SD = 1.825; ES = 0.365) with a statistically significant reduction (*p* < 0.0005) of 10.20 days from the baseline (CI 95%: 8.77; 11.63). In addition, after 1 year of treatment (T12), the use of these symptomatic drugs figured a drastic reduction such that a statistically significant (*p* < 0.0005). Figure 2 is reported the conversion from medication overuse (MOU) to non-medication overuse.

Similar to the clinically and statistically significant reductions analyzed previously, treatment with Erenumab was associated with clinically significant reductions of disability by the MIDAS control tool assessed at times T0, T3, and T12. Overall, at time T12, the score reduction from baseline was 88.32 (*p* < 0.0005) (CI 95%: 68.49; 108.15).

Furthermore, to build a predictive model on the effectiveness of therapy, patients were categorized as super responders when the reduction in migraine days per month was greater than 75% and as responders when the reduction in migraine days per month was greater than or equal to 50%. In the sample taken into analysis, 9 patients were found to be responders and 16 patients were found to be super responders. Utilizing a multivariate logistic regression model, shown in Table 4, it was possible to identify which variables were related to a higher probability of being a super responder.

When the β coefficients of the model are positive, it means that the probability of success increases, on the contrary when they are negative. In our case, the coefficients of MMD and MOU at time T0 are statistically significant. Particularly as MMD increases at time T0, there is an increase in the probability of success and therefore of being categorized as a super responder (b2 = 0.412; *p* = 0.031), while there is a decrease in MOU (b3 = −4.672; *p* = 0.025). No significant influences were found with the variables age, anxiety and/or depression and MIDAS score at time T0, suggesting there is a greater probability of being super responder in patients presenting more severe symptoms in terms of migraine days per month at baseline. Whereas there is a greater probability of failure and not to obtain a response ≥75%.

## 4. Discussion

This real-life analysis was conducted on a heterogeneous population. Chronic migraine was present in 76% of the patients while 24% had an episodic migraine. The number of days of migraine per month was reduced as well as the consumption of symptomatic drugs and the quality of life improved. At baseline the mean frequency of days with migraine pain was 15.24 per month; after 1 year of treatment lowered to 3.36 with a reduction of 11.88 days (*p* < 0.0005). According to previous data, Erenumab was confirmed to have a rapid onset and sustained efficacy over time, which resulted, from a clinical perspective, in a significant gain in migraine pain-free days. A high response rate was achieved, despite the chronicity of the disease, previous conventional treatment failures, and the various comorbidities found in the patients that attended the treatment. The continuous treatment was effective not only in reducing the number of days of migraine pain but also in switching chronic migraine into episodic migraine in all cases. Therefore, it is necessary to emphasize how the possibility of effective therapy can improve not only the prognosis of the patient but also the quality of life. A further analysis concerns the subgroup of patients who previously overused symptomatic drugs. This attitude represents a problem in migraine management. This phenomenon occurs when medications for acute pain are taken with high frequency. This condition is often associated not only with the loss of efficacy of the symptomatic medications themselves but also with the onset of MOU. At time T0 the average number of days per month of use of these drugs was 12.80 while at time T12 was 2.6 with a significant reduction (*p* < 0.0005) of 10.20 days. The reduction in the number of days of migraine crises leads consequently to a reduction in the use of symptomatic drugs, obviating the side effects of their use. In this case, the conversion from medication overuse to non-medication overuse was 100%. The clinical relevance of Erenumab is also confirmed by the improvement in patients’ quality of life and reduction in disease-related disability. This advantage in terms of disease control had a concrete implication on relevant aspects to the migraine patient. In addition, multivariate logistic regression identified some factors related to treatment response. It was found that the presence of MOU reduced the probability of achieving a response ≥75%. Consequently, it is important to highlight how in clinical practice patient identification and education could help to prevent the development of the disorder.

One of the major limitations is the scale of the study. As a single-center study with a limited sample size, we do not claim to define the success of the therapy, but we hoped that our data could be in agreement with those produced globally. However, the strength of the present analysis includes a remarkably longer follow-up of 12 months as compared with previous studies.

## 5. Conclusions

In the real-life analysis, all the prefixed endpoints have been reached and excellent results have been obtained. The confirmation of the validity of CGRP as a therapeutic target represents an extraordinary opportunity for the field of anti-migraine medicine in accordance with what has already been demonstrated by clinical trials. This is because living with migraine is particularly difficult as it is characterized by recurrent periods of darkness highly debilitating imposed by the acute phases of the disease. The patient, therefore, in addition to the high price paid in terms of physical prostration, is forced to safeguard his working, social and relational life organization. Until a year ago migraine was defined as “a pathology that does not kill but does not make you live”, today it remains a disabling disease but finally treatable. Furthermore, the data obtained from the present analysis, which was conducted in a small to medium-sized center, is consistent with data obtained on a large national and international scale. This consideration leads to an important reflection: clinical success is not the result of the size of the center that uses the medicine, but rather the result of a winning methodology that sees the pharmacist in the services as a key figure between the patient and the prescribing doctor in the careful monitoring of therapy from the earliest stage through management of the CNN class, AIFA monitoring registers, collection of clinical and medical data, clinical data collection and pharmacovigilance reports. The pharmacist is not only an expert in medicines, but also the one who dispenses them to the patient and at the same time supports the doctor in the correct pharmacological management, but above all providing specific know-how on the subject of national and international regulatory legislation.

## Figures and Tables

**Table 1:** Profiles of enrolled patients.

	*N*	%
Female	25	96.1
Male	1	3.9
Episodic migraine (EM)	9	34.6
Chronic migraine (CM)	17	65.4
Migraine with aura	7	26.9
Medication overuse	15	57.7
Familiarity	20	76.9

**Table 2:** Prior preventive treatment failure.

	*N*	%
Calcium channel blockers	26	100
Antidepressant	16	61.5
Antiepileptics	18	69.2
β-blockers	5	19.2
Botulinum toxin	2	7.7
5HT antagonists	7	26.8

**Table 3:** Baseline clinical parameters.

	Mean	Standard Deviation	Standard Error
Monthly migraine days (MMD)	15.24	5.07	1.01
Monthly acute migraine medication days	12.80	4.32	0.86
MIDAS	94.72	49.63	9.93

**Table 4:** Multivariate regression analysis.

Variables	B	S.E	d.f.	*p*	or	Lower Confidence Limit	Upper Confidence Limit
Age	0.037	0.057	1	0.521	1.038	0.655	1.661
MMD at T0	0.412	0.191	1	0.031	1.510	1.119	2.007
MOU	−4.672	2.089	1	0.025	0.009	0.001	0.737
Anxiety and/or depression	−1.321	1.391	1	0.342	0.267	0.018	1.314
Score MIDAS at T0	0.017	0.017	1	0.320	1.017	0.555	1.490

MMD: Monthly migraine days; MOU: Medication Overuse; MIDAS: Migraine Disability Assessment Score Questionnaire B: regression coefficient S E: Standard Error; d.f: degrees of freedom Or: Odds Ratio.

## Data Availability

Data and material are available from the corresponding author.
